# Microplastics and viruses in the aquatic environment: a mini review

**DOI:** 10.3389/fmicb.2024.1433724

**Published:** 2024-07-03

**Authors:** Xiuwen Wang, Kaixin Zheng, Yi Wang, Xin Hou, Yike He, Zhiyun Wang, Jiabo Zhang, Xiaochen Chen, Xianhua Liu

**Affiliations:** ^1^School of Environmental Science and Engineering, Tianjin University, Tianjin, China; ^2^The Eighth Geological Brigade, Hebei Geological Prospecting Bureau, Qinhuangdao, China; ^3^Marine Ecological Restoration and Smart Ocean Engineering Research Center of Hebei Province, Qinhuangdao, China; ^4^College of Environment and Safety Engineering, Fuzhou University, Fuzhou, China

**Keywords:** microplastics, viruses, adsorption, mechanism, impact

## Abstract

Microplastics (MPs) have been widely found in the environment and have exerted non-negligible impacts on the environment and human health. Extensive research has shown that MPs can act as carriers for viruses and interacts with them in various ways. Whether MPs influence the persistence, transmission and infectivity of virus has attracted global concern in the context of increasing MPs contamination. This review paper provides an overview of the current state of knowledge regarding the interactions between MPs and viruses in aquatic environments. Latest progress and research trends in this field are summarized based on literature analysis. Additionally, we discuss the potential risks posed by microplastic-associated viruses to human health and the environmental safety, highlighting that MPs can affect viral transmission and infectivity through various pathways. Finally, we underscores the need for further research to address key knowledge gaps, such as elucidating synergistic effects between MPs and viruses, understanding interactions under real environmental conditions, and exploring the role of biofilms in virus–MPs interactions. This review aims to contribute to a deeper understanding on the transmission of viruses in the context of increasing MPs pollution in water, and promote actions to reduce the potential risks.

## 1 Introduction

Microplastics (MPs), as an emerging pollutants, has garnered significant global attention due to their ubiquitous presence and uni-negligible risks. MPs are defined as plastic fibers, fragments, and films with a diameter of less than 5 mm (Thompson et al., 2004; Hartmann et al., 2019). MPs can be categorized into primary MPs, directly manufactured by industries, and secondary MPs, which are derived from the degradation of larger plastic fragments (Cole et al., 2011; Wang et al., 2021a). They are capable of long-distance migration aided by hydrodynamic forces and atmospheric transport, leading to widespread distribution and contamination in remote regions. MPs have been detected in various water bodies, including oceans (Cole et al., 2011), freshwater systems (Li et al., 2018), and domestic wastewater (Zhang et al., 2022a). The distinctive properties of MPs, including large specific surface area, pronounced hydrophobicity, and robust adsorption capacity, render them ideal ecological niches for a diverse range of microorganisms (Zettler et al., 2013). Upon adherence to MPs surfaces, microorganisms secrete a wide variety of extracellular polymeric substances (EPS) (Webb et al., 2009). EPS serves as a protective shield against physical and chemical stresses, such as sand abrasion and photodegradation (Wang et al., 2021b). This protective mechanism promotes microbial colonization and facilitates the formation of biofilms on the MPs surfaces. These biofilms situate at the interface between the plastic surface and the surrounding environment, being commonly referred to as the “plastisphere” (Zettler et al., 2013).

Viruses are widely present on Earth and are critical components of ecosystems and human health. Aquatic environments harbor highly abundant and diverse viruses that outnumber the total number of cellular organisms. The transmission of viruses has led to the emergence of numerous epidemic diseases, such as influenza A (H1N1), SARS, Middle East respiratory syndrome, COVID-19 (Sabar et al., 2022; Yang et al., 2022; Zamhuri et al., 2022). Viral transmission occurs through diverse mechanisms, including direct contact and indirect transmission by environmental substrates (water, air, and food) and various other vectors (Armanious et al., 2016; Yamada et al., 2020; Wang et al., 2023a). Consequently, investigations on the viral transmission mechanisms and factors influencing their virulence have garnered significant concerns.

The adsorption characteristics of MPs and the formation of plastisphere indicate that MPs can serve as artificial “microbial reefs” in aquatic habitats (Bayo et al., 2020). Li et al. (2018) conducted an assessment of virus diversity and potential risks associated with MPs collected from the Beilun River, identifying a total of 1719 different viruses present in the MPs. Additionally, the interaction between MPs and viruses can increase virus virulence through various mechanisms, leading to increased mortality rate of virus-infected organisms (Deng et al., 2021; Seeley et al., 2023). Studies also revealed that co-exposure to SARS-CoV-2 and MPs enhances SARS-CoV-2 infection (Zhang et al., 2022b). However, the precise mechanisms and influencing factors for the microplastic-mediated viral infection remain unclear and warrant further investigation.

In a word, interactions between MPs and viruses in the aquatic environment are an area of emerging research, and there is a considerable lack of knowledge on them. Therefore, this review aims to address the following aspects: (1) conducting a quantitative analysis of the existing literature in this field to summarize the latest progress and research trends; (2) examining the interaction mechanism between MPs and viruses and influencing factors in aquatic environments; (3) suggesting future research directions in this field.

## 2 Literature analysis

**Figure 1:**
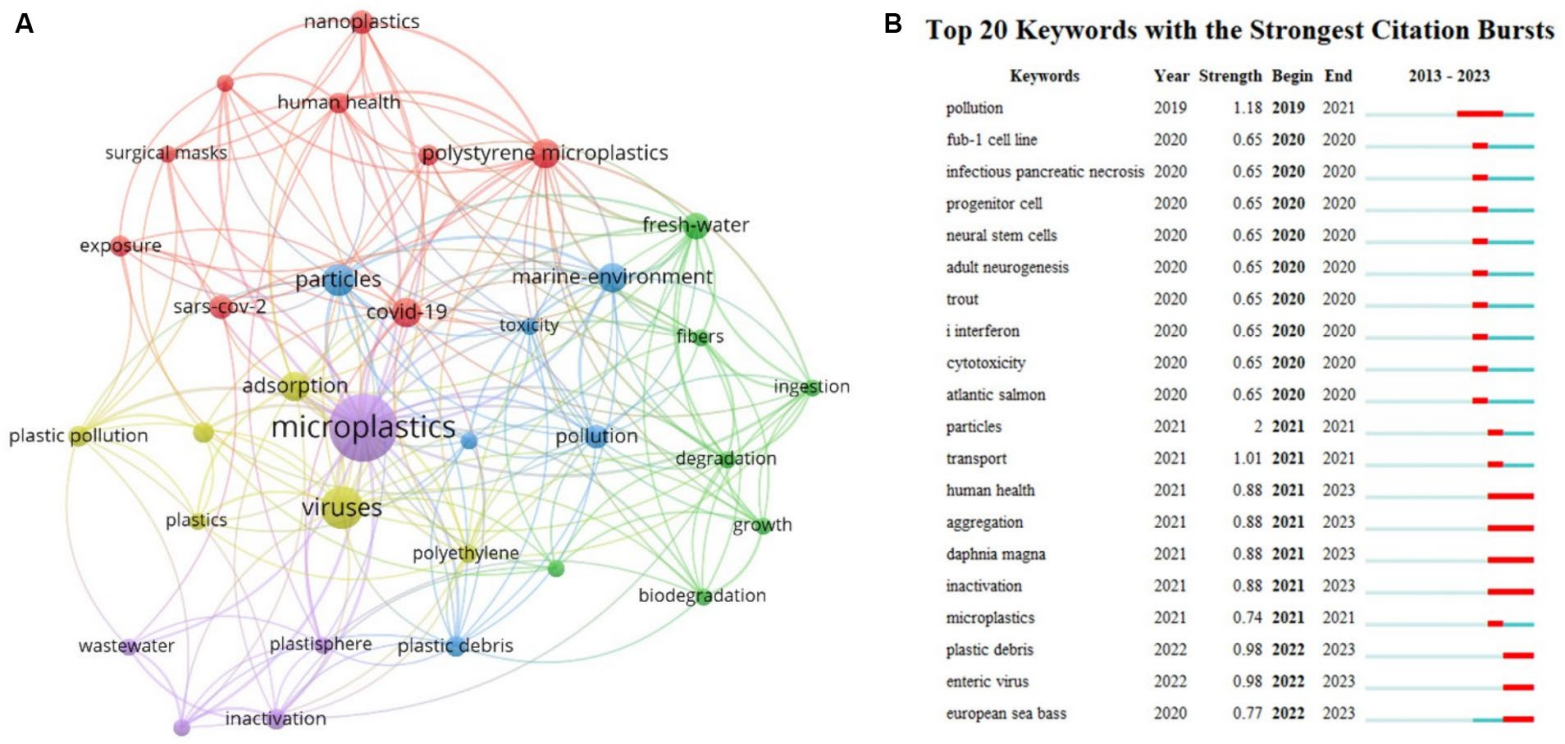
**(A)** Keyword co-occurrence network diagram. **(B)** Keyword burst analysis. 1. Keywords refer to emergent keywords. 2. Year indicates the year when the keyword emerged. 3. Strength represents the prominence intensity of the keyword, with higher values indicating greater influence. 4. Begin and End denote the start and end time of the keyword’s prominence.

Bibliometric analysis was conducted to gain a comprehensive understanding of research trends and hotspots in this field. A detailed literature search was performed in the Web of Science core collection database by using the search formula TS = (microplastic* OR nanoplastic*) AND TS = (*virus*) for the period between January 1, 2014, and January 1, 2024. A total of 170 articles were retrieved. After careful screening, 52 articles were identified as relevant to the research field. These screened articles were used as the input dataset for the subsequent quantitative analysis by using CiteSpace. Before 2020, this research field caught fewer concern and there are only 2 publications during 2019–2020. From 2021, the number of publications kept increasing, reaching 28 in 2023. The increased concern may be attributed to the sudden outbreak of COVID-19 pandemic and the wide use of face masks. The global concern on public health prompted researchers to investigate the interaction between viruses and MPs. Figure 1A shows the keyword co-occurrence network, where the size of the nodes corresponds to the frequency of the respective keyword. Keywords such as particles, marine environment, adsorption, and COVID-19 show high frequencies. This phenomenon suggests that the COVID-19 outbreak has raised concerns about the interaction between MPs and viruses, and the marine environment is a current focus. Figure 1B shows the 20 emergent keywords generated by using Citespace. The red line in the figure represents the time interval during which these emergent keywords garnered significant attention from researchers. For instance, the sudden appearance of “human health” in 2021 indicates that human health issues emerged after the outbreak of the COVID-19 pandemic in 2020. The emergence of keywords such as “trout,” “Atlantic salmon,” “*Daphnia magna*,” and “European sea bass” suggests that these animals serve as important models for studying the mechanisms of MPs-mediated viral toxicity. Furthermore, the emergence of “inactivation” indicates the growing concern on the public health, which facilitates the development of efficient remediation techniques.

## 3 Adsorption of viruses by MPs

### 3.1 Adsorption mechanism

Several studies have demonstrated the robust adsorption properties of MPs towards viruses. For example, Lu et al. (2022) reported a high adsorption rate of 98.6% of the T4 bacteriophages by polystyrene (PS). There are generally two mechanisms: electrostatics and hydrophobic effect governed interactions, and plastisphere-mediated interactions. Viruses can be adsorbed onto the abiotic surface of MPs through non-specific electrostatic interactions (Lu et al., 2022) and hydrophobic forces (Dika et al., 2015). Polyethylene terephthalate (PET) and polypropylene (PP) have been observed to adsorb viruses predominantly through non-ionic forces (Gassilloud et al., 2007), which are directly influenced by the medium pH and the isoelectric point of the virus. Plastisphere plays an important role in the process of virus adsorption onto MPs. Bacteria in plastisphere can produce a diverse array of EPS, enhancing the adhesion and the aggregation of viruses and MPs (Rummel et al., 2017; Moresco et al., 2021). Niu et al. (2023) found that microplastic biofilms selectively enrich certain phages, and Reeves et al. (2023) supposed the association between SARS-CoV-2 transmission and the formation of viral plastisphere in sewage. Moreover, viruses attached to biofilms on microplastic surfaces exhibit increased virulence (Moresco et al., 2021). However, the current understanding on the interaction mechanisms remains limited, necessitating further study.

### 3.2 Influencing factors

The adsorption of viruses by MPs is influenced by various factors. These factors can be classified into three main categories: the aquatic environment, the physicochemical properties of MPs, and the properties of the viruses themselves.

#### 3.2.1 Aquatic environment

Various factors within the aquatic environment can influence the adsorption of viruses on MPs. Water flow rate can directly impact the adhesion of viruses to solid surfaces. Solution pH and ionic strength can affect electrostatic interactions between viruses and MPs. Salinity can affect the ability of bacterial cells to aggregate or form biofilms (De Tender et al., 2015; Oberbeckmann et al., 2018). Dissolved organic matter (DOM) can hinder the interaction between non-enveloped viruses (e.g., MS2 phages) and sorbent surfaces (Armanious et al., 2016). Factors such as water temperature and oxygen content can indirectly affect the bacterial colonization on MPs, thereby impacting the adsorption of MPs to viruses (De Tender et al., 2015).

#### 3.2.2 Physical and chemical properties of MPs

The adsorption of viruses by MPs is directly influenced by the physical and chemical properties of the MPs. Different types of MPs exhibit significant variations in their ability to adsorb viruses, including variations in size, functional group type, surface roughness, zeta potential and aging degree, etc. Lu et al. (2022) found that the adsorption rate of *E. coli* bacteriophage T4 on MPs rose with higher microplastic concentrations but declined with larger MPs sizes. Moreover, MPs functional groups exhibit varying affinities, leading to differences in their adsorption capacities for different viruses. For instance, the presence of a benzene ring in PS enables it to interact with SARS-CoV-2 RNA fragments, forming π–π bonds that potentially modulate the interaction affinity (Zhang et al., 2022a). Zeta potential plays a crucial role in the adsorption of particles by affecting the electrostatic adsorption of viruses onto the MPs surfaces (Savaji et al., 2014). The ultraviolet (UV) aging of MPs was found to enhance the adsorption of viruses due to changed surface zeta potential, increased surface roughness (Dika et al., 2015), and increased hydrophilicity (Wang et al., 2020).

#### 3.2.3 Viral characteristics

Both the surface composition (e.g., proteins, lipids, and polysaccharides) and structure (e.g., viral capsid, tail, and envelope) of the virus can influence its affinity and binding capacity to the surface of MPs (Nobrega et al., 2018; Yamada et al., 2020). Moresco et al. (2022) conducted a study where the non-enveloped SA11virus and the enveloped Phi6 virus were cultured together with PE. They found that the SA11 remained more stable than the Phi6 due to the lack of capsule structure of SA11 promote its binding to the biofilm colonizing MPs. In contrast, the enveloped Phi6 phages experienced rapid envelope dissolution, leading to virus inactivation. Consequently, viral characteristics can determine different virus–plastisphere interactions, which in turn affect the stability and dissemination of viruses themselves.

## 4 MPs affect the transport, survival, and virulence of the viruses

**Figure 2:**
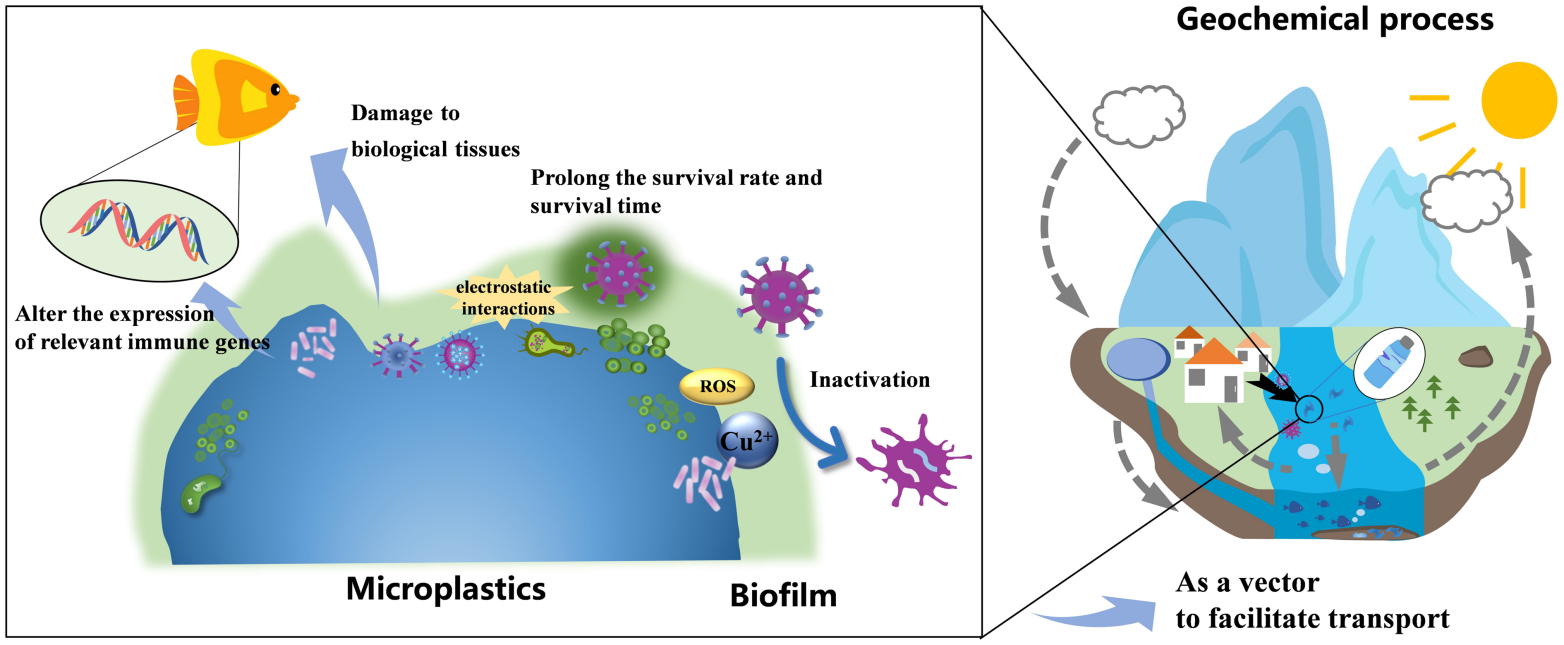
Schematic of the mechanism of microplastics affecting viral biotoxicity in the aquatic environment.

Several studies have demonstrated that MPs can facilitate the dissemination of viruses and exacerbate their biological toxicity. Figure 2 show the proposed mechanisms through which MPs affect the transport, survival and virulence of the viruses.

### 4.1 Act as vectors to facilitate viral transport

MPs can adsorb viruses through electrostatic interactions and formation of surface biofilms, thereby creating a favorable habitat for viruses and serving as efficient carriers for their migration. The formation of surface biofilms alters the buoyancy and viscosity of MPs in water, promoting the long distance transport of viruses via MPs (Bhagat et al., 2021; Li et al., 2022). Besides, the small size of MPs makes them highly prone to ingestion by organisms (Prata et al., 2020). The viruses attached to these MPs can thus easily enter the animal bodies, increasing the infection rate of the virus. This, in turn, can contribute to the emergence of infectious diseases and an escalation in pathogen prevalence (Seeley et al., 2023). For instance, during the COVID-19 pandemic, MPs generated from the decomposed human-used masks may harbor the SARS-CoV-2 virus, prolonging the human exposure to SARS-CoV-2 (Shukla et al., 2022).

### 4.2 Prolong the survival rate and time of the viruses

Several studies (Lu et al., 2022; Moresco et al., 2022) have shown that compared to viruses present in water, viruses harbored in MPs biofilms exhibit longer survival time. Furthermore, these biofilm-associated viruses demonstrate increased stability and a greater capacity to induce biotoxic effects. For instance, more than half of the viruses harbored on the surface of PS MPs remain being detectable even after 10 days, significantly longer than those of free virus particles suspended in water (Lu et al., 2022). Microorganisms attached on MPs may have a higher activity stimulated by dissolved organic carbon leached from plastics (Romera-Castillo et al., 2018). Additionally, microplastic biofilms can offer protection against environmental stressors (Rummel et al., 2017). These “shelters” enable an extended survival time and an increased survival rate of viruses. Furthermore, MPs can form stable complexes with viral genetic fragments, extending the persistence of DNA or RNA fragments within the virus (Zhang et al., 2022b). Zhang et al. (2022a) employed molecular dynamics simulation to investigate the interaction between MPs and viral RNA fragments from SARS-CoV-2, SARS-CoV-1, and HBV. They revealed that MPs can effectively bind to SARS-CoV-2 RNA fragments, leading to the prolonged existence of viral RNA in environment. It is important to note that the type of MPs and viruses, as well as environmental factors can influence the mechanism and intensity of these interactions.

### 4.3 Alter the expression of immune-relevant genes

MPs have been shown to impact immune-related enzyme activity and alter the expression levels of immune-related genes (Liu et al., 2019). This, in turn, compromises the immunity of organisms and leads to increased mortality and infection rates (Liu et al., 2023). Brandts et al. (2021) discovered that MPs have immuno-modulatory effects on juvenile European sea bass (*Dicentrarchus labrax*). Similarly, Limonta et al. (2019) found that after exposure with high-density PE and PS MPs, adult zebrafish showed altered expression of immune-related genes, resulting in a weakened defense ability against pathogens. Liu et al. (2019) also reported a significant decrease in the expression of immune-related genes in blood cells with increasing exposure to MPs. Furthermore, viruses bound to MPs can increase expression of inflammation-related genes and increase viral infectivity (Zhang et al., 2022b).

### 4.4 Damage to biological tissues

MPs ingested by aquatic animals can accumulate in their gastrointestinal tracts, leading to blockages and injuries (Lusher et al., 2013; Bhuyan, 2022). These physical damages can also happened in other biological tissues (Abarghouei et al., 2021). Hamed et al. (2021) found exposure to MPs led to histopathological damage in the kidneys, liver, pancreas, and gills of *Oreochromis niloticus*. Tissue damage can compromise the organism’s immune barrier, heightening susceptibility to viral infections (Zhang et al., 2018). Seeley et al. (2023) demonstrated that simultaneous exposure of fish to viruses and MPs significantly escalates mortality rates compared to exposure to viruses alone. In addition to leading to causing direct physical damage, MPs can also provoke inflammation in biological tissues (Zhang et al., 2023). Various mechanisms have been proposed for this inflammatory response. For instance, once MPs enter into cells, they can induce oxidative stress by generating reactive oxygen species (ROS), consequently triggering inflammation (Cao, 2023). Alternatively, inflammation may be indirectly induced by microbial dysbiosis provoked by MPs (Jin et al., 2018; Wang et al., 2023b). However, further investigations are needed to more comprehensively elucidate these mechanisms.

### 4.5 Inactivation of the viruses

Most studies reported that MPs enhanced the virulence and infectivity of viruses in the aquatic environment. However, there are also studies reporting that MPs exposure can lead to virus inactivation under specific conditions. For instance, Gassilloud and Gantzer (2005) observed the inactivation of poliovirus type 1 through surface adhesion to hydrophobic PP containers. Moresco et al. (2022) investigated PE MPs particles bound to viruses and found that enveloped viruses may undergo solubilization by the biofilm on the MP surface. Furthermore, the elevated concentration of copper ions within the MP surface may increase ROS production (Borkow and Gabbay, 2005). These ROS can impact viral stability, degrade the viral genome and alter the morphology of viral particles (Warnes et al., 2015). These studies is important for developing efficient virus inactivation treatments to control transmission of viruses. Further investigation is needed to find efficient inactivation methods and to understand involved mechanisms.

## 5 Conclusions and prospects

This paper presents a comprehensive overview of the complex interactions between MPs and viruses in the aquatic environment. Electrostatic forces and the hydrophobic effect dominated the interactions. However, these effects are modulated by various factors, including the aquatic environment, the physicochemical properties of the MPs and the characteristics of the viruses. Viruses can adhere to the surface of microplastic particles, potentially altering their transport, fate, and persistence in the environment. Viruses are protected against inactivation factors when bound with the MPs biofilm, and the stability provided by the microplastic environment can potentially enhance the persistence and transmission of viruses in aquatic ecosystems. Once viruses are adsorbed onto MPs, their virulence and transmissibility can be altered through diverse mechanisms. Most of studies reported that MPs tend to enhance the virulence of viruses in the aquatic environment, however, some studies have found MPs can deactivate viruses under some specific conditions, suggesting interaction mechanisms warrants further investigation.

Although researchers have conducted extensive research in this field, there is still a significant gap in our understanding of them. To address these knowledge gaps, it is imperative to prioritize the following issues for further investigation:

1. There is a noticeable dearth of research on the synergistic effects of MPs with viruses. A substantial efforts are needed to unveil how viruses adhere to the surface of microplastic particles, and how these attachments can alter their transport, fate, and persistence in the aquatic environment.
2. The existing research findings primarily concentrate on specific viruses present on plastic objects under laboratory conditions. It is worth noting that the real environmental conditions are more complicated and the interactions between virus and MPs are influenced by various other factors.
3. Biofilms can offer more surfaces for viruses to stick and multiply, but there aren’t many thorough studies in scientific literature about how viruses interact with microplastic biofilm, especially EPS.
4. Many involved mechanisms need to be further elucidated, such as the detail relationships among viruses, water, MPs, and biofilm under different environmental conditions, and how nutrients and energy flow within the microplastic ecosystem? Answering all of these questions requires sophisticated testing methods and the participation of researchers from multiple disciplines.
5. MPs serve as reservoirs for viruses could pose risks to human health if they enter the food chain. Consumption of contaminated seafood or water could potentially expose individuals to viral pathogens associated with MPs. Therefore, the public and policymakers need to be aware of these risks, and develop preventive measures.

## Author contributions

XW: Data curation, Investigation, Writing – original draft, Formal analysis, Methodology. KZ: Investigation, Writing – original draft, Methodology. YW: Investigation, Writing – original draft, Methodology. XH: Investigation, Writing – original draft, Methodology. YH: Investigation, Writing – original draft, Methodology. ZW: Resources, Writing – review & editing. JZ: Resources, Writing – review & editing. XC: Resources, Writing – review & editing. XL: Conceptualization, Funding acquisition, Supervision, Writing – review & editing.
